# Road Traffic Noise and Annoyance: A Quantification of the Effect of Quiet Side Exposure at Dwellings

**DOI:** 10.3390/ijerph10062258

**Published:** 2013-06-03

**Authors:** Yvonne de Kluizenaar, Sabine A. Janssen, Henk Vos, Erik M. Salomons, Han Zhou, Frits van den Berg

**Affiliations:** 1 TNO, Department of Urban Environment and Safety, P.O. Box 49, Delft 2600 AA, The Netherlands; E-Mails: sabine.janssen@tno.nl (S.A.J.); henk.vos@tno.nl (H.V.); erik.salomons@tno.nl (E.M.S.); han.zhou@tno.nl (H.Z.); 2 Public Health Services (GGD), Municipality of Amsterdam, P.O. Box 2200, Amsterdam 1000 CE, The Netherlands; E-Mail: fvdberg@ggd.amsterdam.nl

**Keywords:** road traffic noise, environmental noise, transmission modeling, exposure assessment, quiet side, noise annoyance

## Abstract

Previous studies indicate that residents may benefit from a “quiet side” to their dwellings. The influence of the level of road traffic noise exposure at the least exposed side on road traffic noise annoyance was studied in Amsterdam, The Netherlands. Road traffic noise exposure was assessed at the most and least exposed façade (*L_den,most_* and *L_den,least_* respectively) of dwellings for subjects in a population based survey (N = 1,967). It was investigated if and to what extent *relative quietness* at the least exposed façade affected the level of road traffic noise annoyance by comparing two groups: (1) The subgroup with a relatively quiet façade; (2) the subgroup without a relatively quiet façade (large *versus* small difference in exposure between most and least exposed façade; DIF ≥ 10 dB and DIF < 10 dB respectively). In addition, it was investigated if and to what extent *L_den,least_* affected the level of road traffic noise annoyance. Results indicate a significantly lower road traffic noise annoyance score at a given *L_den,most_*, in the subgroup with DIF ≥ 10 dB *versus* DIF < 10 dB. Furthermore, results suggest an effect of *L_den,least_* independent of *L_den,most_*. The estimated size of the effect expressed in an equivalent change in *L_den,most_* approximated 5 dB for both the difference between the two subgroups (DIF ≥ 10 dB and DIF < 10 dB), and for a 10 dB change in *L_den,least_*.

## 1. Introduction

Exposure to environmental noise has been associated with a broad range of health effects. Of these effects, annoyance is the most widely recognized and is considered the most prevalent. In a recent study, the WHO concluded that sleep disturbance and annoyance form the main burden of disease from environmental noise in Europe [1]. Road traffic is an important cause of noise annoyance in urban areas. For traffic noise annoyance, exposure-response relationships have been established based on pooled analyses of a large international database [2]. In an EU position paper, these relationships have been recommended to be used for estimating the expected prevalence of traffic noise annoyance [3]. The expected prevalence of annoyance is predicted from levels of exposure at the most exposed façade of dwellings. However, locally the actual prevalence of noise annoyance may substantially differ from predicted values (see e.g., [4]). This difference may be explained by a broad range of (area specific) characteristics of both the population and the physical environment.

One of these characteristics is the exposure at the least exposed side of dwellings. People living in dwellings with a (relatively) quiet least exposed side may be expected to be better off than average. Similarly, inhabitants of dwellings with relatively high noise exposure at multiple sides may be expected to be worse off than average. It has been previously hypothesized that access to a quiet side may reduce the adverse effects of noise by offering an “escape” from the noise to the inhabitants, e.g., by providing the option to spend time or sleep at the quiet side of the dwelling [5,6].

Previous studies indeed indicate that having access to a (relatively) quiet side is associated with a comparatively lower annoyance [7,8,9]. Different approaches to investigate the influence of exposure at the least exposed side have been followed. For example, Öhrström *et al.* defined a quiet side as an exposure level at the least exposed façade below 45 dB *L_Aeq,24h_*, while other studies have looked at the influence of a relatively quiet façade, expressed as an indicator for difference between the exposure at the most and the least exposed side [7]. To date, however, only a limited number of studies is available. More studies are needed to further corroborate the hypothesized effect, and to enable the comparison of results between studies in different populations. Furthermore, there is a need for further quantification of the influence of exposure at the least exposed side. In this study, the influence of the level of road traffic noise exposure at the least exposed side on annoyance was studied in a population based survey in Amsterdam, the Netherlands. Road traffic noise annoyance was available on an 11 point scale for a large urban population. The effect of exposure at the least exposed side was investigated in two ways. First, it was investigated if and to what extent *relative quietness* at the least exposed façade affected the road traffic noise annoyance level. Second, it was investigated if and to what extent the road traffic noise level at the least exposed side (*L_den,least_*_, _continuous) affected the level of road traffic noise annoyance, in addition to the level at the most exposed side (*L_den,most,continuous_*).

## 2. Methods

Road traffic noise exposure at both the most and the least exposed façade of dwellings (*L_den,most_* and *L_den,least_*, respectively), was estimated by model calculations for all addresses in the city of Amsterdam. Noise exposure was linked to questionnaire data on self-reported traffic noise annoyance and potential confounding factors of subjects in a population based survey. In this way a substantially sized sample with both noise exposure and response data was obtained. This provided the opportunity to investigate the hypothesized association between exposure at the least exposed side of the dwelling on the annoyance response of the inhabitants.

### 2.1. Study Population

Survey data were collected in 2008 by the Public Health Service (GGD) of the Municipality of Amsterdam (Amsterdam Health Monitor 2008) [10]. The following procedure was used: First, a random sample of 13,600 inhabitants of Amsterdam was drawn from the municipal population register. From this initial sample, subjects who had moved out of the study area or had died were excluded. Secondly, the sample was stratified by age and by district, to ensure comparable representation of all age groups and all urban districts (city boroughs) of Amsterdam. Two different versions of the questionnaire were developed: one version was dedicated to the 16 to 54 years age group, and one version for the older age group (aged 55 years and over). The annoyance question was available only in the questionnaire for the age group of 16 to 54 year olds. This sub sample consisted of 6,800 subjects, who were invited to participate. Addresses were available only for respondents who had indicated that their data could be used for further studies. The response rate was approximately 50%. Thus, road traffic noise exposure at the dwelling façades could be estimated for 1,967 subjects.

Survey data were collected by a postal questionnaire or by an internet questionnaire, or (if requested) with the aid of an interviewer. The main purpose of the survey was to gauge the health status of the Amsterdam adult population, including demographic, socioeconomic, psychosocial and environmental determinants. The survey included questions on self-reported noise annoyance from a number of sources (apart from road traffic noise including e.g., noise from neighbors and humming noise (e.g., from fans)) and a broad range of potential confounders including socio-demographic variables (e.g., age, gender, and education level). Data on education was available in four categories: low (primary education), medium low (lower professional and intermediate general education), medium high (intermediate professional and higher general education), and high education (higher professional education and university).

Data on road traffic noise annoyance were available from the following questions in the questionnaire: “Thinking of the last 12 months, when you are at home, which number on a scale from 0 to 10 best represents to what extent you are being annoyed or disturbed by noise from the following sources”, followed by: (a) traffic on roads with a maximum speed limit greater than 50 km/h, and (b) traffic on roads with a maximum speed limit of 50 km/h. From these two road traffic noise annoyance questions, an annoyance scale was constructed by taking for each respondent the maximum score of both items. It was also possible to indicate that either type of traffic noise was not audible.

### 2.2. Noise Exposure

Exposure to road traffic noise was determined by model calculations for all dwellings (addresses in the population registry) in the city of Amsterdam. For each dwelling, exposure levels were calculated at the most and least exposed façade (*L_den,most_* and *L_den,least_*, respectively). Here, *L_den_* is the day-evening-night level, which is a “weighted average” of the levels *L_day_* for the day period (7:00–19:00 h), *L_evening_* for the evening period (19:00–23:00 h), and *L_night_* for the night period (23:00–7:00 h), and includes “penalties” of 5 and 10 dB for the evening and night periods, respectively. The model calculations were performed with the Dutch standard calculation method for road traffic noise (SRM2) [11]. This method is a standard engineering method, which is also used for strategic noise mapping in the framework of the Environmental Noise Directive 2002/49/EC (END) [12]. Input for the model calculations includes: geometrical data of buildings and noise barriers, geometrical data of roads (location and surface type of road segments), traffic data for all road segments (vehicle intensities, traffic composition (incl. light vehicles (passenger cars), medium-heavy vehicles, and heavy vehicles), and driving speed), and geometrical data for land surface types. The data were provided by the municipality of Amsterdam, for the year 2011. Road traffic data were available for the urban roads with substantial traffic intensities in Amsterdam (typically with a traffic intensity above about 1,000 vehicles per 24 h). Noise levels were calculated depending on the height of the dwelling.

For the most exposed façade, noise levels below 45 dB were recoded as 45 dB. The level of 45 dB was assumed an approximate representation of ambient noise in urban areas. This value has previously been used as a cut off value for the most exposed façade, e.g., in the development of the exposure response curves for annoyance [2,4]. For the least exposed façade, levels below 40 dB were recoded as 40 dB, which was assumed an approximate representation of ambient noise at the quiet side in urban areas.

### 2.3. Statistical Analyses

Linear regression analyses were performed to investigate the relationship between road traffic noise exposure at the most exposed façade, and at the least exposed façade, and the annoyance score (scale 0 to 10). Exposure at the least exposed façade was entered in the model two ways: First, the effect of a relatively quiet façade was investigated (difference between most and least exposed façade (DIF < 10 dB *versus* DIF ≥ 10 dB)). Second, the influence of the road traffic noise level at the least exposed façade (*L_den, least_*, as a continuous variable) was investigated. Linear regression analyses were carried out for 3 models: (1) Unadjusted model; (2) Adjusted model (adjustment for age, gender and education); (3) Full model: as (2) with additional adjustment for annoyance from noise by neighbors and humming sounds (e.g., fans), as these sources possibly disrupt quietness at the least exposed façade. All analyses were carried out with the statistical software package IBM SPSS statistics version 20.0.0.

## 3. Results

### 3.1. Characteristics of the Study Population

**Figure 1:**
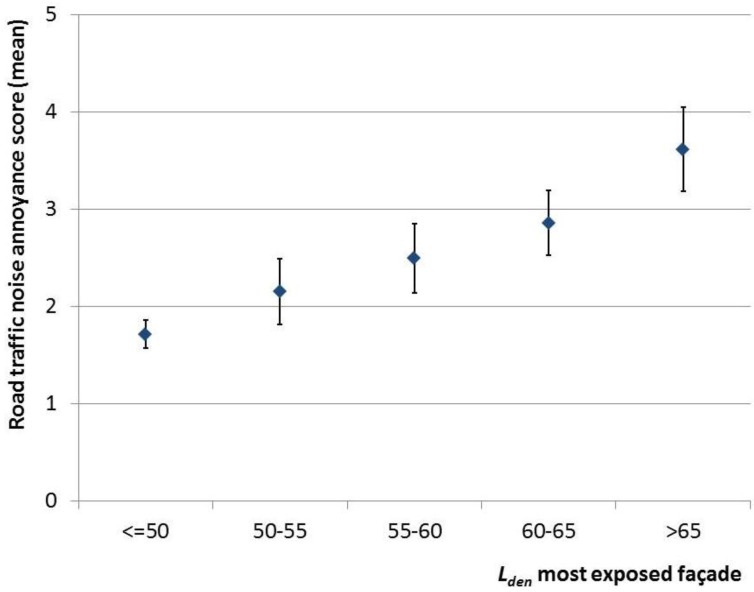
Mean road traffic noise annoyance score (scale 0 to 10), with 95% confidence intervals for the Amsterdam study population.

Table 1 shows the characteristics of the Amsterdam study population. The average age of the study population is approximately 36. Relatively more women than men participated in the study, and the percentage of subjects with high education level is comparatively high with slightly over 50%. The average reported road traffic noise annoyance score (scale 0 to 10) in the Amsterdam study population is approximately 2. The average annoyance from neighbor noise exceeds this average with a mean score of approximately 3. Figure 1 shows the mean road traffic noise annoyance score, for categories of *L_den,most_* with confidence intervals, for the Amsterdam study population.

**Table 1:** Characteristics of the Amsterdam study population.

Variable	
Age (average, SD)	35.8 (10.4)
Men (%)	38.7
Women (%)	61.3
Education low (%)	6.9
Education medium low (%)	17.3
Education medium high (%)	25.2
Education high (%)	50.6
Annoyance road traffic noise scale 0 to 10 (average, SD)	2.2 (2.7)
Annoyance noise neighbors scale 0 to 10 (average, SD)	2.9 (2.9)
Annoyance noise humming sound (e.g., fans) scale 0 to 10 (average, SD)	1.3 (2.3)
*L_den,most_* (average, SD)	52.3 (8.0)
*L_den,least_* (average, SD)	41.7 (3.7)
Relatively quiet façade (DIF ≥ 10 dB; %)	40.5

### 3.2. Relatively Quiet Façade and Annoyance

**Figure 2:**
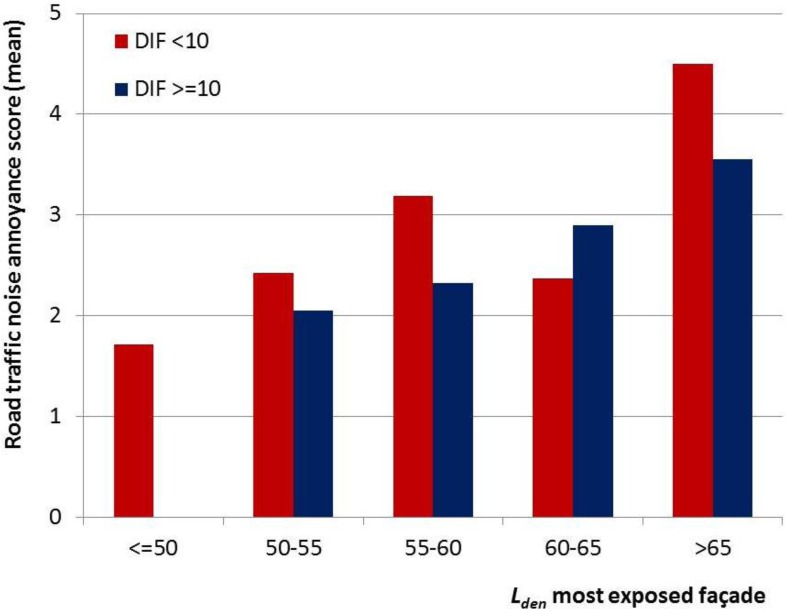
Mean annoyance score (scale 0 to 10) in the Amsterdam study population for two categories of difference between most and least exposed façade (DIF < 10 dB *versus* DIF ≥ 10 dB), using 5 dB intervals of *L_den,most_*.

Figure 2 presents the mean annoyance score for the subgroup with a relatively low difference between most and least exposed façade (DIF < 10 dB) and the subgroup with a relatively high difference between most and least exposed façade (DIF ≥ 10 dB), by most exposed façade road traffic noise level (*L_den,most_*). The figure suggests the mean annoyance scores are lower for the subgroup with a relatively quiet façade, although not consistently for all categories of *L_den,most_*.

Table 2 provides an overview of the results from the linear regression analyses. In the analyses, the annoyance score is predicted from the exposure at the most exposed façade (*L_den,most_*; dB; continuous) and the availability of relative quietness at the least exposed façade (difference between *L_den,most _*and *L_den,least_* in two categories (DIF < 10 dB *versus* DIF ≥ 10 dB)), with additional adjustment for covariates in the 2 extended models. Table 2 shows there was a significant association between road traffic noise annoyance and the availability of relative quietness at the least exposed façade, with significantly lower annoyance in a situation with a relatively high difference as compared to a relatively low difference in exposure at the most and least exposed façade. The table shows that the parameter B estimates remain significant and similar in magnitude between the 3 models, indicating that the effect did not diminish after additional adjustment. Adjusted R Squared of Model 1, 2 and 3 are 0.055, 0.055 and 0.214 respectively. The adjusted R Squared of these same models, but without relative quietness, are slightly lower, with 0.053, 0.053 and 0.212 respectively. The interaction between *L_den,most_* and relative quietness was tested. However, this was found to be not significant (results not shown).

**Table 2:** Parameter estimates for the contribution of *L_den,most_* (dB, continuous) and the difference between *L_den,most_* and *L_den,least_* (DIF ≥ 10 dB *versus* DIF < 10) in the linear regression model for annoyance score.

	B_Model 1 _(SE)	B_Model 2_ (SE)	B_Model 3_ (SE)
*L_den,most_*	0.101 (0.013) ***	0.100 (0.013) ***	0.099 (0.012) ***
DIF ≥ 10	−0.463 (0.205) *	−0.448 (0.207) *	−0.481 (0.190) *

Model 1: Unadjusted model; Model 2: Adjusted for age, gender, education; Model 3: Full model: Adjusted for age, gender, education, annoyance from neighbor noise and humming noise (e.g., fans). Statistical significance is indicated as usual: * p < 0.05; ** p < 0.01; *** p < 0.001. The numbers B (SE) are the unstandardized regression coefficients, with their standard error (in brackets).

**Figure 3:**
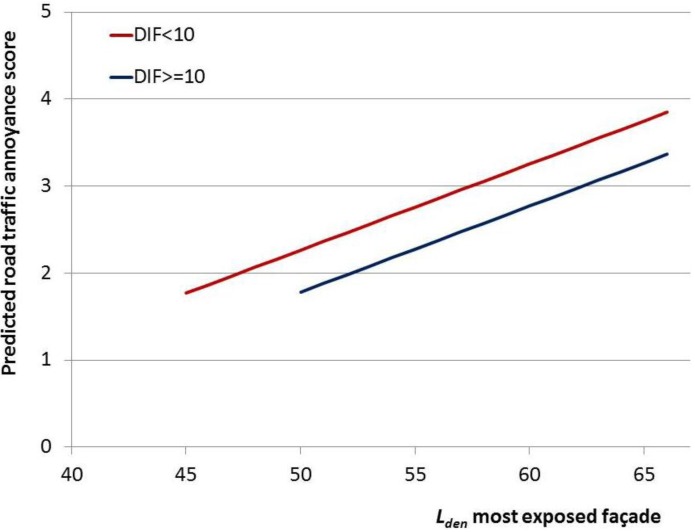
Road traffic noise annoyance score predicted from *L_den,most_* (dB, continuous) and difference between *L_den,most_* and *L_den,least_* in two categories (DIF < 10 dB *versus* DIF ≥ 10 dB). Results from linear regression analyses.

In Figure 3 regression lines are shown for the two subgroups of most and least exposed façade exposure difference (DIF < 10 dB *versus* DIF ≥ 10 dB), for the full model. Results are visualized for the assumption of equal gender distribution, average age, most prevalent education level, and average annoyance score from neighbor noise and humming noise (e.g., fans). The parameter B estimate for *L_den,most_* determines the slope of the regression line, while the parameter B estimate for the difference determines the additional change in annoyance score related to this difference. The horizontal shift in the regression lines, which can be read from the figure by looking at equal annoyance scores, provides an estimate of the difference in “effective noise level” between these two groups. The figure shows that the horizontal shift in the regression lines approximates 5 dB, *L_den,most_*.

### 3.3. Exposure at the Least Exposed Façade and Annoyance

Table 3 shows the results from linear regression analyses where the annoyance score is predicted from the exposure at the most exposed façade (*L_den,most_* in dB; continuous) and the least exposed façade (*L_den,least_* in dB; continuous). A significant association was found between *L_den,least_*, and annoyance score in the full model. This model adjusted for age, gender, education, and annoyance from neighbor noise and humming noise (e.g., fans), independently of *L_den,most_*. Adjusted R Squared of Model 1, 2 and 3 are 0.054, 0.054 and 0.213 respectively. In addition, the interaction between *L_den,most_* and *L_den,least_* was tested. However, this was found to be not significant (results not shown).

**Figure 4:**
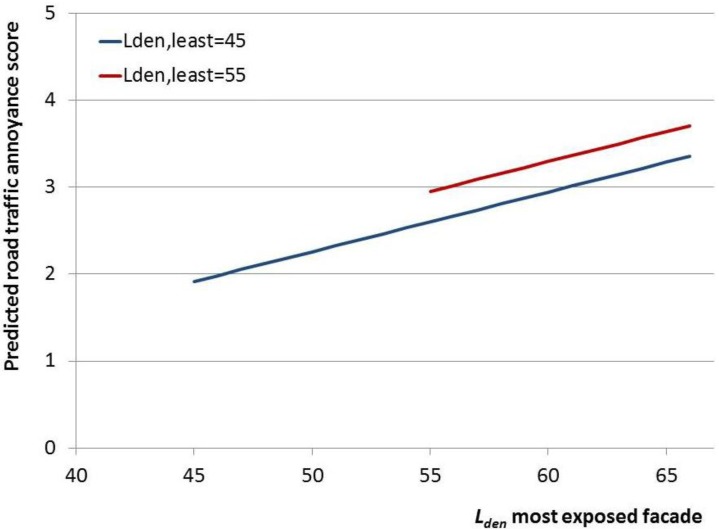
Road traffic noise annoyance score predicted from *L_den,most_* (dB, continuous) and *L_den,least_* (dB, continuous). Results from linear regression analyses.

In Figure 4, the regression lines are visualized for various levels of *L_den,least_*, for the full model. The results indicate that for the predicted annoyance score a reduction of 1 dB at the least exposed façade corresponds to a reduction of approximately 0.5 dB at the most exposed façade (*i.e.*, a 5 dB horizontal shift (*L_den,most_*) in regression lines corresponds to a 10 dB change in *L_den,least_* (e.g., from *L_den,least_* 45 dB to *L_den,least_* 55 dB)).

**Table 3:** Parameter estimates for *L_den,most_* (dB, continuous) and *L_den,least_* (dB, continuous) in the linear regression model for annoyance score.

	B_Model 1 _(SE)	B_Model 2_ (SE)	B_Model 3_ (SE)
*L_den,most_*	0.073 (0.008) ***	0.073 (0.008) ***	0.069 (0.007) ***
*L_den,least_*	0.026 (0.017)	0.025 (0.018)	0.035 (0.016) *

Model 1: Unadjusted model; Model 2: Adjusted for age, gender, education; Model 3: Adjusted for age, gender, education, annoyance from neighbor noise and humming noise (e.g., fans). Statistical significance is indicated as usual: * *p* < 0.05; ** p < 0.01; *** p < 0.001. The numbers B (SE) are the unstandardized regression coefficients, with their standard error (in brackets).

## 4. Discussion and Conclusions

The results of this study indicate that there is an association between road traffic noise annoyance and both the availability of *relative* quietness at the least exposed side of dwelling, and the actual exposure level at the least exposed façade itself (*L_den,least_*), independent of *L_den,most_*. The road traffic noise annoyance score (at a given exposure level at the most exposed façade) was lower in the group with *relative* quietness at the least exposed façade, expressed as an exposure difference between most and least exposed façade (DIF ≥ 10 dB). Similarly, results suggest lower annoyance with decreasing noise level at the least exposed façade (*L_den,least_*). An association between *L_den,least_* and annoyance, independent of *L_den,most_,* indicates not only that *lower* exposures at the least exposed façade may be better for the inhabitants, it also implies that *higher* exposures at the least exposed façade may *increase* adverse effects. In addition, it should be noted that even at *low* values of *L_den,least_*, adverse effects are still to be expected when *L_den,most_* is high. Similarly, the results on *relative* quietness only indicate a difference in expected road traffic noise annoyance score between the groups with a DIF < 10 dB compared to DIF ≥ 10 dB. However, also at a DIF ≥ 10 dB, at high façade levels (e.g., *L_den,most_* = 71 dB, *L_den,least_* = 60 dB), exposures are still undesirably high, and on the bases of existing exposure response curves (see e.g., [1]), still adverse health effects are to be expected (even though people might be (slightly) better off than in a situation with an exposure of e.g., 71 dB on multiple sides). Nevertheless, these results provide further support for the hypothesis that inhabitants exposed to road traffic noise may benefit from a quiet side to the dwelling.

These results are in line with previous studies. Öhrström *et al.* previously investigated the potential benefit of a quiet side to the dwelling amongst 956 individuals aged 18–75 years, within the Soundscape Support to Health research program [7]. They studied the relationship between having access to a quiet side of the dwelling (defined as a façade with an *L_Aeq,24h_* ≤ 45 dB) and a number of adverse noise effects, including annoyance. Results indicated that having access to a quiet side corresponded to a decrease in disturbances by an average of 30–50%. This decrease was estimated to correspond to a reduction in noise level at the most-exposed side of about 5 dB (*L_Aeq,24h_*). Likewise, De Kluizenaar *et al.* studied the association between road traffic noise and the environmental noise annoyance response within two groups: the subgroup with a relatively quiet façade (difference in road traffic noise level between the most and least exposed façade > 10 dB *L_den_*), and the subgroup without a relatively quiet façade (difference < 10 dB) [8]. Results suggested annoyance to be less likely in the group with a relatively quiet façade. The recent study of Van Renthergem and Botteldooren provides additional support for a beneficial effect of the presence of a quiet façade at a dwelling [9]. This study showed that the absence of a quiet façade (expressed as a difference in road traffic noise level between the most and least exposed façade < 10 dB) leads to a substantial increase of self-reported noise annoyance.

Further and indirect evidence for the potential benefit of a quiet side came from studies that investigated the difference in noise response between respondents having a bedroom facing the traffic source, or facing the noise shielded side. In a study on road traffic noise annoyance and sleep disturbance in a stratified random sample of 1,000 respondents, Bluhm *et al.* reported a lower prevalence of both self-reported road traffic noise annoyance and sleep disturbance for respondents with their bedroom facing a “quiet side” (defined as: not facing the street) [13]. Amundsen *et al.* estimated the benefit of having the bedroom facing the noise-shielded side of the dwelling on noise annoyance to correspond to a 6 dB noise reduction [14]. In other words, the difference in annoyance between “having the bedroom on the least-exposed façade” *versus* “having the bedroom on the most-exposed façade” was estimated to correspond to an exposure difference of 6 dB in outdoor noise level (*L_Aeq,24h_*) at the most exposed façade. In line with these findings Gidlöf-Gunnarsson *et al.*, based on field study data obtained for 1,695 respondents, reported a twice as high prevalence of general noise annoyance among residents in dwellings with a balcony/patio oriented towards the railway, and about 1.5 times higher for residents with their bedroom facing the railway [15]. Furthermore, Lercher *et al.* reported on the ALPNAP-study, where they found a clear trend of reduced risk of hypertension for participants with their bedroom facing a quiet yard [16]. In addition, Selander *et al.* found that risk estimates for myocardial infarction were particularly elevated for participants annoyed by noise mostly in their bedroom [17]. The above results indicate a possible mechanistic pathway through disturbance of sleep, and/or the importance of exposure at the least exposed side (assuming that on average, particularly at higher exposure levels, people tend to sleep at the quiet side if they have the option).

In addition to access to a quiet side as such, the visual and functional quality of the quiet side has been suggested to have an influence. In a previous study, Gidlöf-Gunnarsson and Öhrström studied the influence of the physical environmental quality (degree of naturalness and utilization) of “quiet” outdoor courtyards (defined as *L_Aeq,24h_* ≤ 48 dB, façade reflex included) in a sample of 385 residents [18]. They found that access to a “high quality” quiet court yard was associated with less noise annoyance among the residents. Furthermore, the results of an earlier study by the same researchers have suggested that “better” availability to nearby green areas may decrease the risk of annoyance [19]. Thus, all of the above factors may affect the perceived quality of the least exposed side, and potentially may (positively or negatively) affect its benefit. In the “Quiet Places Project” in Amsterdam, De Booij and Van den Berg also reported the potential importance of the presence of vegetation and other pleasant stimuli, in addition to relative quietness of a place, based on a survey among 809 respondents [20].

Some limitations should be noted. Since this is an observational study with a cross sectional design, the possibility that a related factor other than road traffic noise was in fact responsible for the observed effect, in theory cannot be fully ruled out. However, as the investigated effect is specifically road traffic noise related, and the least exposed side exposure influences the overall road traffic noise exposure, a causal relationship is plausible. Secondly, while road traffic noise may be assumed to be the dominant source of environmental noise in urban areas, the possibility for inhabitants to “escape” from the noise to a quiet side of the dwelling will also depend on the noise from other sources, which may harm the relative quietness at the back side of a dwelling. Indeed, previous research has shown that noise generated by installations (e.g., fans) at the quiet side of dwellings can cause substantial annoyance and thereby “spoil” the quiet [21]. Similarly, noise from neighbors may affect the “quietness” at the least exposed side. In view of both issues raised above, in this study, we tried to minimize the risk of confounding by adjustment for a number of potential confounders, including education (as an indicator of socio-economic status), age and gender, as well as the influence of noise from other sources: neighbors and installations (humming noise e.g., fans). However, it should be noted that other potentially important modifying factors, about which unfortunately no information was available, may influence the effect of the quiet side exposure, such as orientation of the bedroom towards the noise source, noise sensitivity, and the visual quality and accessibility of the quiet side.

The study population consisted of a sample of the total Amsterdam city population. Still, the possibility that the generalizability of results to the Amsterdam city population may to some extent have been influenced by selective response cannot be ruled out. Furthermore, it is not known to what extent the inhabitants of the city of Amsterdam may be representative to the general population in The Netherlands or in Europe. Therefore, it would be valuable to investigate the influence of noise exposure at the least exposed side to confirm results in future studies, also in other cities.

In this study, road traffic noise exposure was calculated with The Netherlands’ standard calculation method for road traffic noise, SRM2, a method that is comparable to other European engineering methods for road traffic noise modeling. These methods may be further optimized for refined exposure assessment at the noise shielded side of buildings [22]. So far however, no such method has (yet) been adopted for noise mapping. Should this limitation have influenced the effect estimate, it may be assumed that the actual association is slightly stronger than found. However, this would need to be confirmed in future studies. Furthermore, road traffic data was available for the urban roads with substantial traffic intensities in Amsterdam (including roads typically with a traffic intensity above about 1,000 vehicles per 24 h). In future studies the exposure assessment may be further refined if traffic intensity data will become available also for the smallest roads in the network. Currently, however, this is typically not available at that level of detail. Nevertheless, our results show a clear association between quiet side indicators and the noise annoyance. In this study both detailed information on road traffic noise annoyance as well as objectively assessed road traffic noise exposure levels were available for a large sample of residents (N = 1,967). Road traffic noise annoyance was available on a scale from 0 to 10 (11 point scale), in line with the international recommendation [23]. The large sample size, together with this detailed information for each resident, may be expected to have increased the power of the analysis.

In large urban areas, access of residents to quietness is important to allow and support restoration needed to recover from the impact of stress caused by daily activities. This is likely to be of particular importance in the immediate living environment, the home. This study provides further support for the hypothesized benefit of quietness at the least exposed façade. Because of the adverse effects of noise exposure on health [1], it is important to avail of practical applicable measures to reduce the impact of exposure for urban residents as much as possible. In urban planning processes, first of all, this needs to be addressed by striving towards low exposure at dwellings in general, starting with the most exposed façade, which still appears to have a higher impact on annoyance. One approach to further improve the noise environment may be the creation of quiet facades to offer an “escape” to the noise for the inhabitants. In existing situations with high exposure levels, particular attention may need to be paid to dwellings with high exposure at multiple sides.
